# Semi-Automated Data Processing and Semi-Supervised Machine Learning for the Detection and Classification of Water-Column Fish Schools and Gas Seeps with a Multibeam Echosounder †

**DOI:** 10.3390/s21092999

**Published:** 2021-04-24

**Authors:** Annalisa Minelli, Anna Nora Tassetti, Briony Hutton, Gerardo N. Pezzuti Cozzolino, Toby Jarvis, Gianna Fabi

**Affiliations:** 1 National Research Council, Institute for Marine Biological Resources and Biotechnology (CNR-IRBIM), largo Fiera della Pesca 1, 60125 Ancona, Italy; annalisa.minelli@irbim.cnr.it (A.M.); annanora.tassetti@cnr.it (A.N.T.); gianna.fabi@cnr.it (G.F.); 2 Echoview Software Pty Ltd., GPO Box 1387, Hobart, Tasmania 7001, Australia; toby.jarvis@echoview.com; 3 SFERANET S.R.L. Via Giulio Vincenzo Bona, 120, 00156 Roma, Italy; gerardopezzuti@gmail.com

**Keywords:** multibeam echosounder, water column imaging, machine learning, fish schools, gas plumes, target detection and classification

## Abstract

Multibeam echosounders are widely used for 3D bathymetric mapping, and increasingly for water column studies. However, they rapidly collect huge volumes of data, which poses a challenge for water column data processing that is often still manual and time-consuming, or affected by low efficiency and high false detection rates if automated. This research describes a comprehensive and reproducible workflow that improves efficiency and reliability of target detection and classification, by calculating metrics for target cross-sections using a commercial software before feeding into a feature-based semi-supervised machine learning framework. The method is tested with data collected from an uncalibrated multibeam echosounder around an offshore gas platform in the Adriatic Sea. It resulted in more-efficient target detection, and, although uncertainties regarding user labelled training data need to be underlined, an accuracy of 98% in target classification was reached by using a final pre-trained stacking ensemble model.

## 1. Introduction

Multibeam echosounders (MBESs) are active sonars and have been historically designed for hydrographic purposes, such as submerged obstacle detection, bathymetry and seabed characterization [1,2,3,4,5,6]. Over the last decade or so, technological advancements in MBES hardware and software have facilitated the use of MBES data for water-column imaging (WCI), which has emerged as viable means of hydrographic data quality control [7,8] and found increasing use in oceanographic studies [9].

Single and split-beam echosounder (SBES) technology existed since at least the 1940s and has been traditionally used for the purpose of the evaluation of fish populations [10]. Nowadays MBES WCI provides numerous opportunities for fisheries and eco-biological research, especially when combined with the ability to acoustically cover a larger volume of the water column. Indeed, the wide area insonified with a single swath (typically covering about 120°) differentiates MBESs from SBES whose main beam widens less than 20° and therefore requires far more survey time to achieve equivalent coverage. Novel and compelling applications of WCI from MBES range from qualitative descriptions of fish school behavior and morphological characteristics [11,12,13,14], oil and gas leakage detection [15,16,17,18], kelp ecosystem mapping [19,20] and aquaculture monitoring [21], to assessing the mean abundance of marine macro-litter [22,23] and providing other valuable insights to marine systems [24,25,26,27].

The interest in WCI measurements is reflected by ongoing implementation of WCI functionality into modern MBES and development of respective powerful online and post processing software packages for hydrographic and fishery applications. Quality Positioning Services Inc. (QPS, Zeist, The Netherlands) progressed with the threshold detection in the FMMidwater module, CARIS allowed the user to utilize WCI to supplement bottom detection results with the release of HIPS 8.0, while Echoview Software Software Pty Ltd. (Hobart, Australia) has continuously added to and improved its suite of tools for MBES data processing since 2001.

Target detection—where a target is considered an object that demonstrates an acoustic impedance discontinuity across its boundary [28]—for MBES WCI data has to date heavily relied on nearly fully supervised and thus time-consuming data processing procedures [9,29,30]. In addition to being mostly manual, WCI data processing strongly depends on the subjective expertise of the operator that has to select thresholds, extract and then classify targets [15,31].

Many experts have worked to improve efficiency and reliability in target detection from MBES WCI [15,32], and recently a few studies on target detection in multibeam WCI used features and classifiers to reduce human interaction requirements and improve efficiency. Feature-based Machine Learning (ML) is indeed able to detect targets in consecutive WCI quickly, in real-time and automatically, and may have a key role especially when we consider that reviewed data sets could act as training for future ML models. Urban et al. [33] advanced automated detection of bubble streams working with their spatiotemporal behavior and a thresholded median signal-based mask applied on Kongsberg EM302 WCI. Zhao et al. [34] used the AdaBoost cascade classifier, combining the Haar-like feature and Local Binary Patterns feature to automate gas plume detection and segmentation by conducting shallow and deep-water experiments using data collected by Kongsberg EM710 and EM122 MBES. In Williamson et al. [35] filtering, detection and tracking using a modified nearest-neighbor algorithm provided robust tracking of diving seabirds, fish and fish schools using an EK60 SBES (Simrad, Kongsberg, Norway) in the vicinity of marine renewable energy installations. Similarly, Cotter and Polagye evaluated the performance of three ML supervised algorithms for automatically classifying marine fauna in MBES data collected from an uncalibrated BlueView M900–2250 (Teledyne, Fredericton, NB, Canada) [31]. For each algorithm, they first used a hill-climbing search to optimize the set of hand-engineered features describing each tracked target (e.g., descriptions of target shape, motion, intensity and additional covariates such as the time of the day).

**Figure 1:**
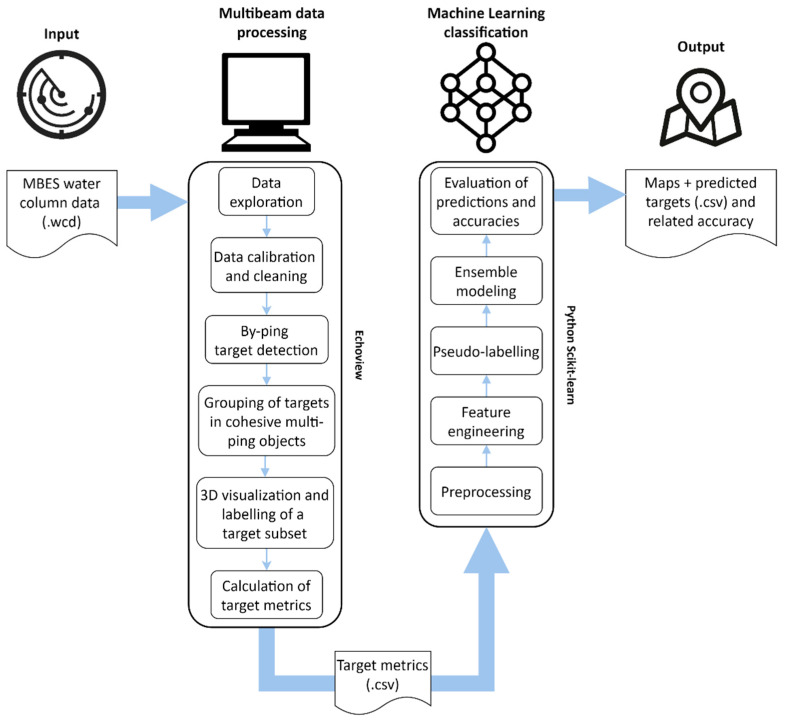
Overview diagram of the full workflow.

In this experimental context a comprehensive and reproducible WCI data processing workflow has been developed from what was presented in Minelli et al. [36], improving the efficiency of target detection and classification in huge volumes of WC data whilst reducing the amount of human interaction required for the above-mentioned operations. Assuming that targets such as fish schools, gas seeps and noise differ in morphological metrics, scattering degree and behaviour in time, the proposed workflow (Figure 1) makes use of Echoview and ML techniques to: (i) speed-up the target extraction procedure by drafting a generalized workflow that can be run automatically in Echoview; and (ii) classify extracted targets using a pre-trained stacking ensemble ML framework.

The method is described and tested on WC data collected from a hydrographic multibeam echosounder. Acquired WCI covers a small area surrounding a gas platform in the central Adriatic Sea, where the presence of gas seeps is well known. Similarly, schools of fish are expected to be frequently detected as targets given the function of the platform as artificial habitat. Results and performance of the approach are discussed, while capabilities and limitations of the uncalibrated MBES in fish school characterization and gas detection are addressed considering the quality of the raw data and their reliability. From this, insights for improving classification of water-column targets in MBES data are given, as well as for the transferability of trained models.

## 2. Materials and Methods

### 2.1. Study Sites

**Figure 2:**
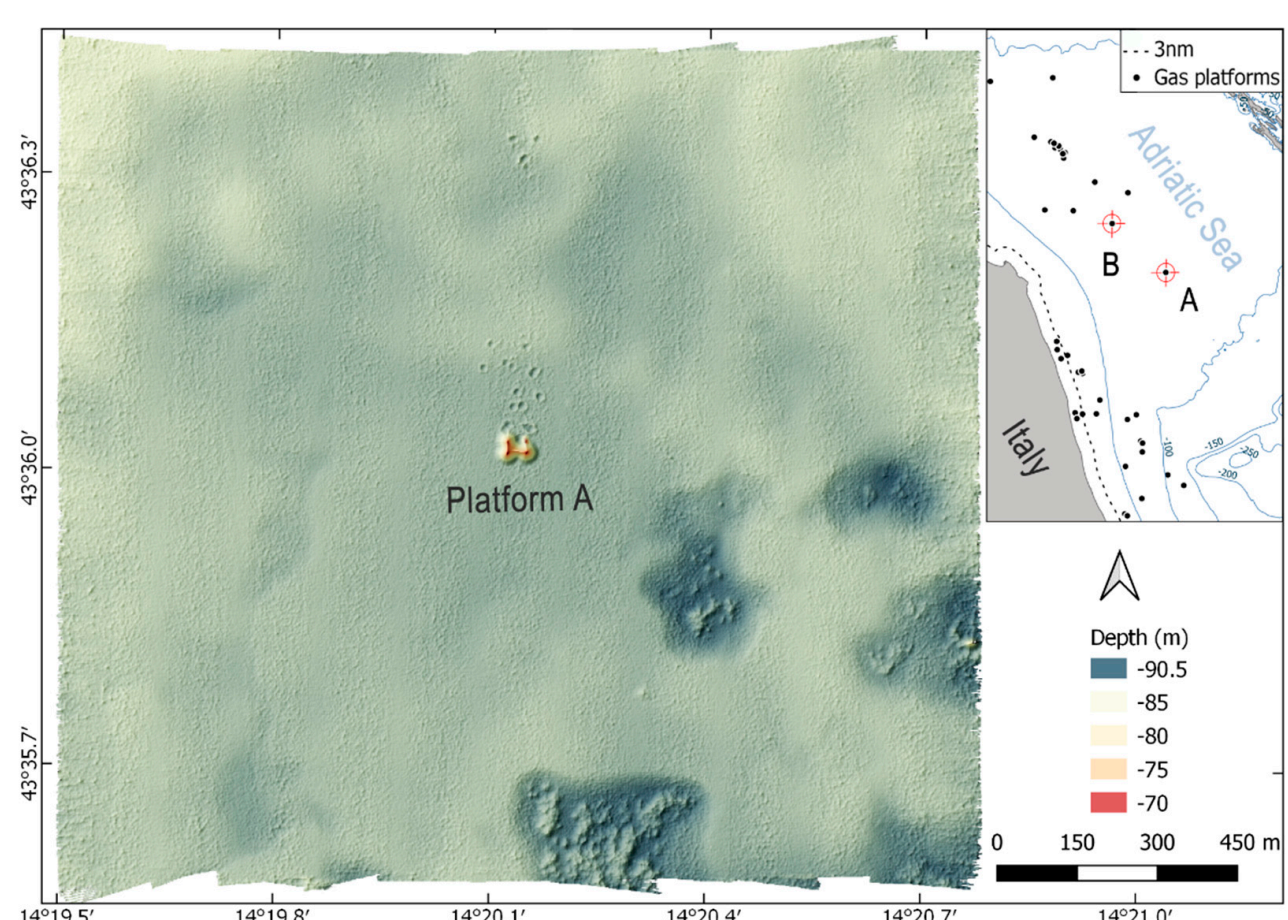
Site A (1.5 km × 1.5 km area surrounding the gas platform A) and related DTM (1 m resolution and 4× vertical exaggeration). The red cross symbols in the locator map highlight the location of two study sites in the Adriatic Sea.

The two study sites in the Adriatic Sea (Figure 2) each consisted of a 1.5 km-square area centred around a four-legged gas platform. The water depth was ~85 m at site A and ~77 m at site B, with the seafloor consisting of muddy sand.

In the south-eastern corner of site A were numerous depressions (pockmarks) in the sediment caused by methane seeps. These seeps are of particular interest because they work counter to ocean carbon capture and sequestration (CCS) schemes by releasing carbon back to the ocean [16]. There were no such depressions at site B.

### 2.2. Multibeam Data Acquisition

Measurements were made with a EM2040CD MBES (Kongsberg, Kongsberg, Norway) from CNR-IRBIM’s 14 m-long research vessel, Tecnopesca II. The EM2040CD is a compact, dual-transducer version of the EM2040 MBES designed primarily for seafloor-echo detection at ranges < 600 m. Each transducer yields a 2D fan (“swath”) of backscatter samples within an array of 400 beams. A number of parameters are configurable by the user within the Kongsberg Seafloor Information System (SIS) software, from which the system determines the number, steering angle, spacing and opening angle of the beams in the swath.

**Figure 3:**
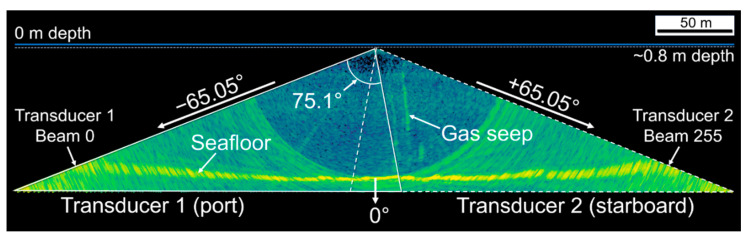
The geometry of the EM2040CD transducer swaths and an example of a gas seep and the seafloor (see Figure 4 for color scale).

The transducers were hull mounted at ~0.8 m depth and the system configured to transmit 600 µs narrowband acoustic pulses centred at ~300 kHz. To avoid interference between transducers, the frequencies were slightly separated, with the difference automatically determined as a function of the pulse duration (shorter pulses have a larger bandwidth, necessitating a greater difference). Following each transmission, the system calculated 256 equiangular receive beams per transducer (nominally 1° along-track by 0.29° across-track per beam), resulting in a 1° along-track by 75.1° across-track swath for each transducer. The transducers were spaced ~0.4 m apart in the across-track plane and oriented to ensure coverage directly below the vessel, yielding a combined across-track swath of 130.1° over 512 beams (Figure 3). Both transducers were synchronised and set to transmit at a rate of 2.6 Hz.

Water sound speed was measured with a miniSVS Sound Velocity Sensor (Valeport, Devon, UK) mounted close to the transducers to enable real-time beamforming and sample-range calculation by the SIS software. The range extent of each sample was 64 cm. The beam steering angles and sample depths were corrected for vessel pitch, roll, heave and yaw in real time using measurements from a Kongsberg Seatex Motion Reference Unit MRU 5 and an Anschütz Gyro Compass Equipment Standard 20 Compact Type 110–222 NG001 (0.02° roll and pitch accuracy, and 0.1° heading accuracy; Raytheon Anschütz, Kiel, Germany).

**Figure 4:**
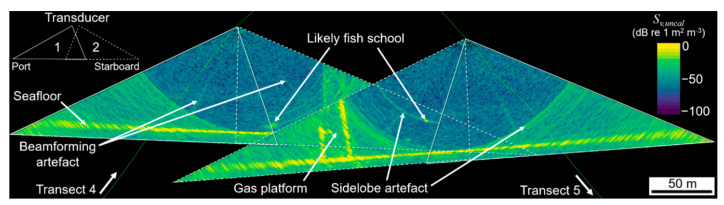
3D oblique view of the transducer swaths from adjacent transects at site A, showing the degree of swath overlap and examples of beamforming and sidelobe artefacts, the gas platform, fish schools and the seafloor.

The seafloor (Figure 3 and Figure 4) was detected in real time from the MBES measurements by the SIS software using the recommended “basic filters” settings, since no significant peaks or pits were expected on the seafloor. MBES samples > 10 m in depth beyond the detected seafloor in each beam were set to “no data”. The vessel location was measured with an SPS855 GNSS Modular Receiver (sampling rate 2 Hz, Trimble, Sunnyvale, CA, USA) and all sensors synchronised to the GPS-measured UTC time. The time-referenced vessel position and attitude, and MBES beam-steering angle, sample range and sample backscattering magnitude for each ping were saved to a series of proprietary-format binary data files with .wcd filename extensions.

Three surveys were conducted at site A and one survey at site B (Table 1), consisting of 10 evenly spaced, parallel transects ~ 1.5 km in length. The transects were run in alternating north-south or east-west directions at ~2 to 2.6 m/s^−1^ (~4 to 5 knots), with the spacing between transects (~100 m) chosen based on the water depth to ensure a minimum of 50% across-swath overlap between adjacent lines (Figure 4). Due to the time that elapsed between subsequent surveys, the state of the transducers was thoroughly checked and maintained for biofouling and deterioration of its components (during regular dry-dock operations), and calibration cycles for MRU and SVS probes were scheduled to meet required specifications.

The MBES data from surveys 1 and 2 are publicly available at https://doi.org/10.17882/79142 (access date 23 April 2021) [37]. These surveys were carried out as part of an ongoing monitoring program by CNR-IRBIM of gas platforms in the central Adriatic Sea since the late 1990s [11,24,38].

### 2.3. Multibeam Data Processing

The SIS-detected seafloor depth and backscatter measurements were processed with CARIS HIPS & SIPS software (version 11.0, Teledyne, Fredericton, NB, Canada), taking into account tide corrections, manual editor and quality control tools. A Digital Terrain Model (DTM) of the seafloor was created as a Combined Uncertainty and Bathymetry Estimator (CUBE) surface with a resolution of 1 m (Figure 2).

**Figure 5:**
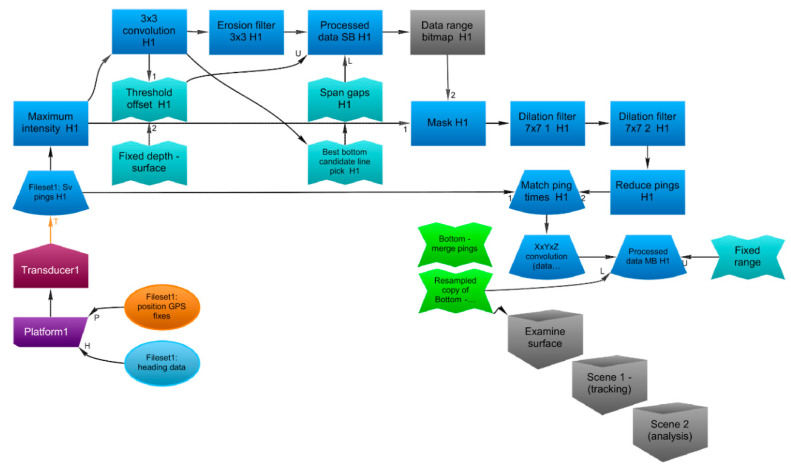
Echoview screenshot of the “Dataflow” window showing the data-processing steps applied to transducer 1 (“H1”). The same steps were applied to transducer 2.

The water-column backscatter measurements were processed with Echoview software (version 11.1.34, Echoview Software Pty Ltd.). Key data-processing decisions are described in Table A1 (Appendix A) and the Echoview “Dataflow” window shown in Figure 5. Echograms and ancillary measurements were visualized and queried to establish general data characteristics.

MBES pings from both transducers were merged into a single variable, from which the bottom depth was automatically estimated for all beams in all pings using Echoview’s surface detection algorithm. This generates a 3D triangulated irregular network (TIN) object that represents the seafloor. The TIN was resampled (2 m North-South and East-West resolution with a maximum triangulation distance of 20 m) to reduce geometric complexity and smooth out any irregularities resulting from noise or other artefacts.

MBES pings were converted to a summarized 2D view of time by range using the Maximum Intensity operator, where each sample (at range R) contains the maximum value of all of the corresponding multibeam samples that are also at range R (sometimes referred to as “stacked beams”). The stacked beam pings were examined, and pings containing unusually high backscatter through the full extent of the data collection range were removed from further analysis. Statistical algorithms of convolution “median” and “erosion” were applied to the remaining pings to remove stochastic noise and reverberation (unwanted backscatter). These two algorithms replace the value of each cell of the echogram with respectively the median and minimum values found in a 3 × 3 sliding window centered on the sample [39].

Other algorithms were applied to automatically identify samples that were:

- Below the minimum range of the seafloor, using the Best Bottom Candidate line pick algorithm which examines windows of pings to identify corresponding backscatter peaks [40];
- above the maximum range of any near-surface entrained air bubbles, using the Threshold Offset algorithm which defines a virtual line representing the nearest occurrence of a specified threshold value with respect to a nominated line in an acoustic variable [41]. Samples above this line were excluded from further analysis.

The remaining samples were then further manipulated using a 3 × 3 dilation filter (where a sample is replaced by the maximum of the eight surrounding samples, [39]) and bitmap operators to highlight and then discard all pings that did not contain any backscatter of interest in the water column.

The original multibeam pings were filtered to only keep the pings that contained backscatter of interest as identified through the 2D echogram process. Successively the samples within those pings were smoothed using, ahead of further processing, a XxYyZ convolution kernel operator (where a sample is replaced by the mean of the surrounding 45 samples, [39]).

**Figure 6:**
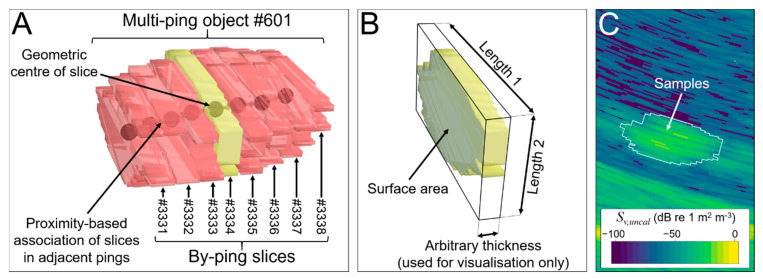
The geometry of slices and multi-ping objects. (**A**) A 3D object such as a fish school or a cluster of bubbles from a gas seep was conceived as a sequence of backscattering cross-sections (“slices”) delineated across multiple pings. A slice detected in one ping was associated with a slice in the next ping (black spheres joined by arrows) based on spatial proximity by applying a tracking algorithm more commonly used for following the movement of an object through space and time. (**B**) 3D view of slice #3334 in (**A**). (**C**) 2D echogram view of slice #3334 in (**A**).

Samples within 4 m of the transducer face or beyond a vertical offset from the resampled TIN were excluded from further processing. An image analysis algorithm referred to as “school detection” in Echoview was applied to identify and delineate contiguous clusters of above-threshold samples that met specified size conditions on a ping by ping basis, which generates a polygon “region” that represents a cross-section through an object (hereafter referred to as a “slice”, Figure 6). Slices may have represented cross-sections through fish schools, gas seeps, the gas platform, or other features that still remained after the data cleaning process (Figure 3 and Figure 4) and may have been partially or completely insonified within the swath. The slices were given an arbitrary along-track thickness of 0.8 m to generate a 3D object (polyhedron) for each slice (Figure 6B) and grouped using Echoview’s “region tracking” algorithm into larger multi-ping objects on the basis of proximity (Figure 6A).

Although region tracking is typically used to characterize the movement of a target through space and time, in this case we used it in a novel way to determine which slices in adjacent pings were part of a larger object. At a ping rate of 2.6 Hz and a vessel speed of <2.6 m s^−1^, we expected the targets of interest (fish schools and gas seeps) to be observed across multiple pings. The grouping of slices into multi-ping objects is a preliminary level of classification.

**Figure 7:**
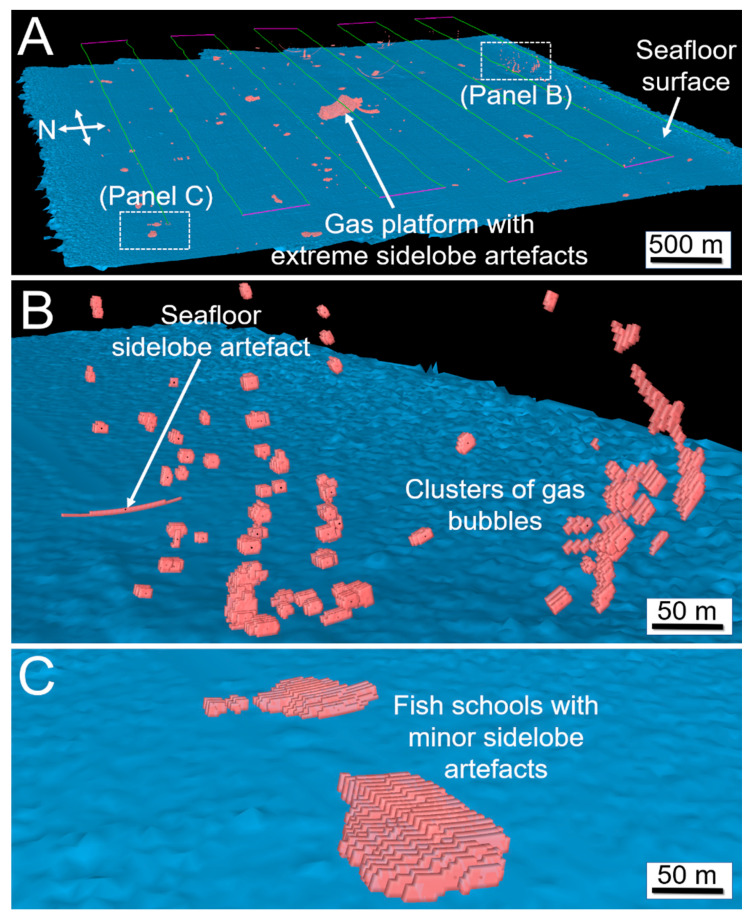
3D visualisation of the slices (red) created from Survey 1. (**A**) Overview of the entire survey. (**B**) Clusters of gas bubbles rising from the seafloor in the SE corner of the survey area. (**C**) Fish schools in the NW corner of the survey area. Sidelobe artefacts are caused by the unwanted detection of strong targets in neighbouring beams, causing the target to appear wider than it really is.

The slices created during Survey 1 are shown in Figure 7. A subset of individual slices was manually reviewed to be used in the machine-learning stage of the analysis, and classified as one of fish school (FISH), gas seep (GAS), platform leg (PLATFORM) or NOISE (e.g., sidelobe artefacts or entrained air bubbles from the vessel) on the basis of their shape, evolution in time and known target occurrence at the site. Labelled slices, consisting of about 8% of the slices in Survey 1, were extracted, stratified by depth and equally portioned among the four classes.

Metrics for all slices, including location, shape, position within the MBES ping, and backscatter properties were characterized and exported to comma-separated value (CSV) files.

### 2.4. Machine Learning Classification

**Figure 8:**
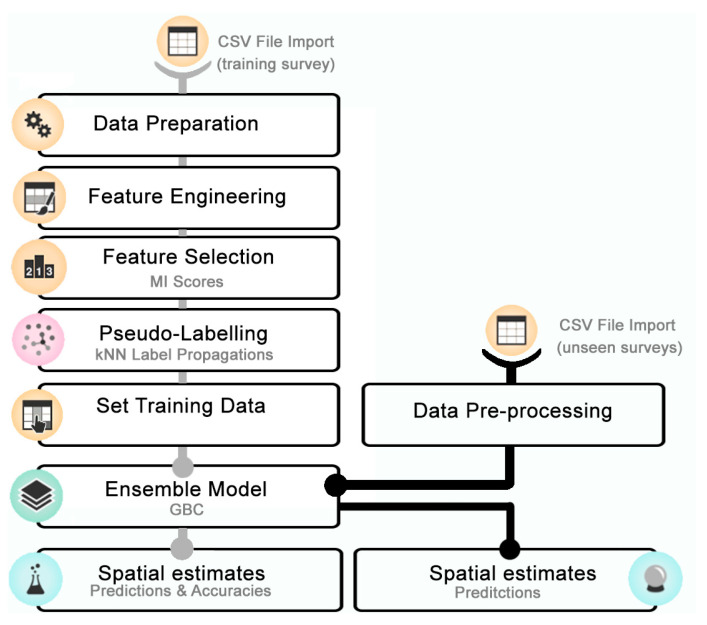
ML data-processing workflow to train the model before performing predictions on unseen data.

The CSV file with the labelled slices (and their calculated metrics) for Survey 1 was first prepared (Figure 8), by: (i) manually retaining only the features (out of the 68 total metrics available) whose relevance was evaluated by common knowledge; (ii) normalizing features to a comparable scale (Yeo-Johnson transformation, [42]), with the exception of coordinates; and (iii) extending labelling within multi-ping objects (see Figure 6a). This last step was due to the assumption that contiguous slices lying on the same track (with the same multi-ping object number) likely belong to the same object and thus to the same target class. An example is reported in Figure 6a where unlabeled pink slices inherited the label (e.g., FISH) that was manually assigned to the yellow slice as belonging to the same multi-ping object. It allowed us to increase the labelled dataset, while reducing operator intervention and saving valuable time.

A feature engineering was then needed to generate new and sensible features, before performing the feature selection on the whole set of features by using Mutual Information (MI) scores [43]. Feature engineering improved the predictive modelling performance on the dataset by transforming its feature space to better differentiate target classes. In particular, coordinates of the geometric centers were flattened into x and y, while four new features were generated as combination between some of the existing metrics:

- Base, given from the ratio between surface area and height of the slice;
- Length ratio, derived from the ratio between the two longest dimensions of the slice;
- Depth ratio, given from the ratio between geometric and mass center depths;
- Sv_UNCAL_ diff, given from the difference in logarithmic scale of maximum and mean values recorded for the same slice.

A last additional feature (Cluster) was identified based on the results of a K-means algorithm that hierarchically clustered data into four groups using Vertices (number of vertices in the polyhedral object that represents the slice), Mass center latitude and Mass center longitude.

The labeled data were then passed to help train the ML procedure with a k-nearest neighbor (kNN) semi-supervised learning called pseudo-labelling [44], that combined both labeled and unlabeled (or better, pseudo-labeled data) to train the following gradient boosting classifier (GBC [45]), resorting to a brute-force grid search to get the optimal hyperparameter setting. The GBC is an ensemble (stack) of weak learners that are combined to minimize the loss, or the difference, between the actual class value (label) of the training data and the predicted class value.

Before training the ensemble model, labeled data were randomly stratified while splitting up into training and testing sets (70:30, respectively) [46] in order to have approximately the same percentage of samples of each target class as the complete set. The feature space was lightened of the raw coordinates that could bias the model when transferred to sites other than Survey 1.

Once evaluated on the testing dataset, the trained model was saved and used to classify the remaining targets of Survey 1 and perform predictions on 3 different unseen datasets (Survey 2, Survey 3, Survey 4). With this last aim, unseen data needed to be preprocessed to fit the feature space the model was trained on.

For more detailed information on the ML pipeline the code is available at: http://doi.org/10.5281/zenodo.4621173 (accessed on 23 April 2021) [47]. It was developed in Python with the support of its Scikit-learn machine learning library [48].

## 3. Results

**Figure 9:**
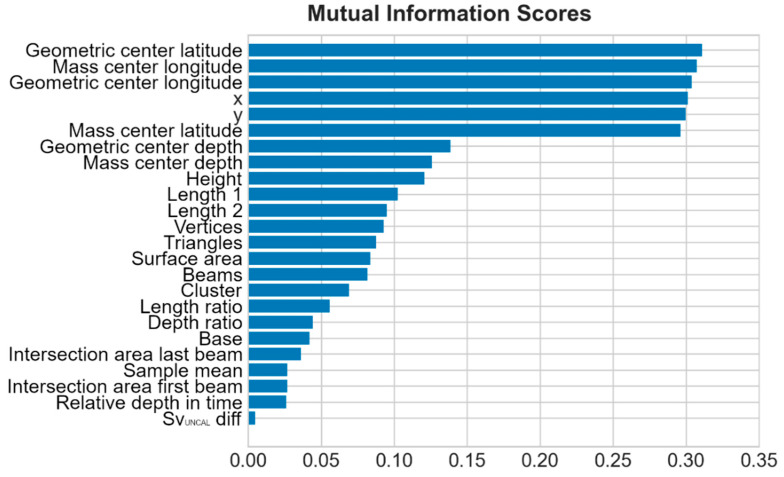
Feature space exploration and MI scores.

**Figure 10:**
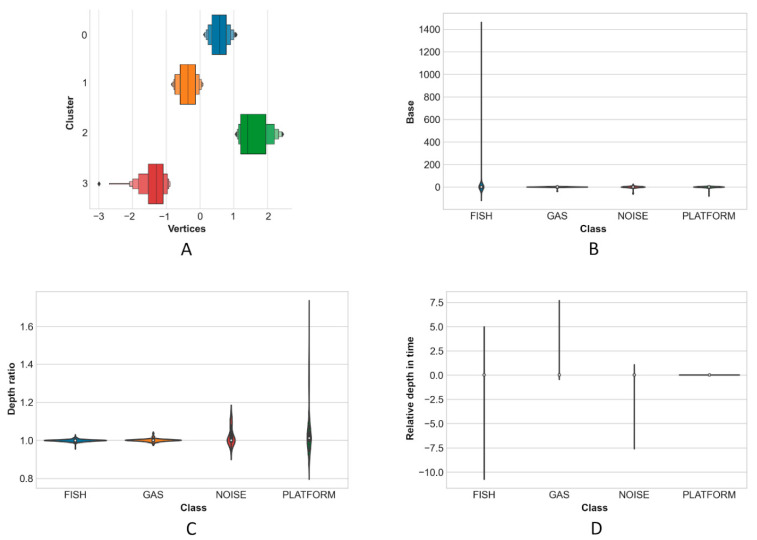
Feature space exploration by kNN *Cluster* and *Vertices* (**A**); by *Base*, describing the shape with low values for oblong targets (**B**); by *Depth ratio* (**C**); by *Relative depth in time* (**D**).

Twenty-three out of the 68 metrics extracted by Echoview were predicted to be relevant (by domain knowledge) to distinguish between target classes, and so manually retained for the ML classification. Afterwards, by feature engineering, the feature space was increased to 27 features, and then reduced to 24 (Table 2) by removing highly correlated features and replacing them with their combinations. Selected features were mutually (Figure 9) and separately (Figure 10) explored to verify their performance in differentiating target classes, before being passed to the kNN for label propagation (Pseudo-Labelling).

The wide distribution of K-means clusters in Figure 10A shows that the derived feature Cluster was relevant in improving target class separability. Base and Depth ratio show their potential to separate, respectively, FISH and PLATFORM targets (Figure 10B,C). Relative depth in time assumes positive values for the rising GAS targets but tends to zero for slices that are attributed to the platform (Figure 10D).

The ratio between the area of a slice and its height (Base) seems to be highly variable only for fish schools, while it tends to zero for oblong targets—labelled as GAS and/or PLATFORM—whose vertical dimension is far greater than the surface area (see also Figure 6 for more information on slice appearance). Moreover, while FISH and GAS targets have their geometric and center of mass depths more or less coincident, the same cannot be said for the platform (and, to a lesser degree, NOISE) whose center of mass is generally at lower depths both for the shape assumed by slices and for the backscatter strength which is generally higher at deeper ranges. This relative shift in mass is probably due to the angle of incidence of the beams relative to the platform, and further exaggerated by an increased side lobe effect on backscatter with range.

To train the model, a dataset composed by 2418 targets was extracted during the WC data processing of Survey 1, taking 5 h to export results from approximately 2 GB of WCI data on a moderately-specified computer (Dell XPS 9570 with Intel i7-8750H @ 2.2 GHz, 16 GB RAM, Samsung PM981 NVMe SSD). 218 slices (8%) were manually reviewed and directly labeled by the operator in Echoview, which then grew to 529 (30%) by automatically labeling slices belonging to the same track.

**Figure 11:**
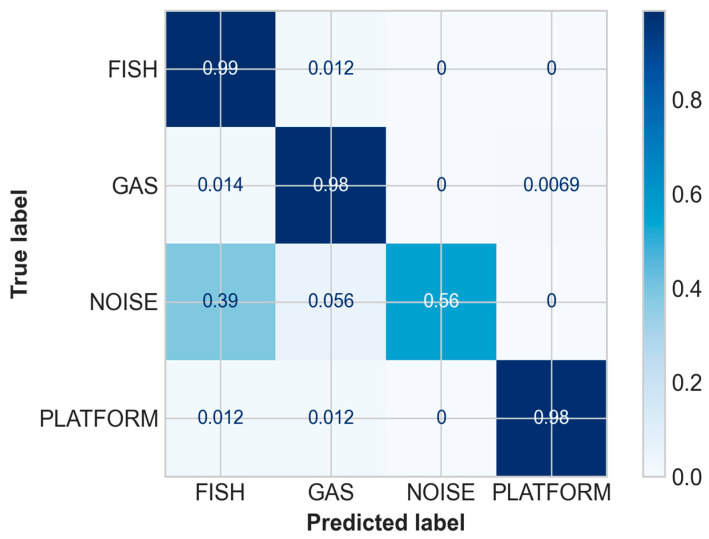
Confusion matrix of the pseudo-labelling.

These 592 labelled slices were used to feed the kNN semi-supervised learning, resulting in high-quality pseudo-labels with an overall accuracy of 96% between target classes, while the proportion of correctly identified targets ranged from 56% for the NOISE class to 99% (maximum) for the FISH class (Figure 11). Additional classification statistics are listed in Table 3.

**Figure 12:**
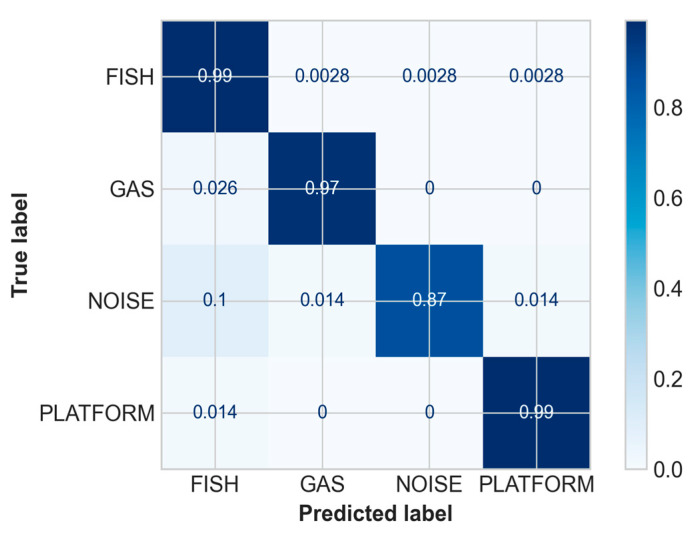
Confusion matrix of the ensemble model.

The overall accuracy of the ensemble prediction method for fish, gas, noise and platform was about 98% (Table 4), while the confusion matrix in Figure 12 provides a more detailed breakdown of correct and incorrect final classifications for each of the four target classes.

**Figure 13:**
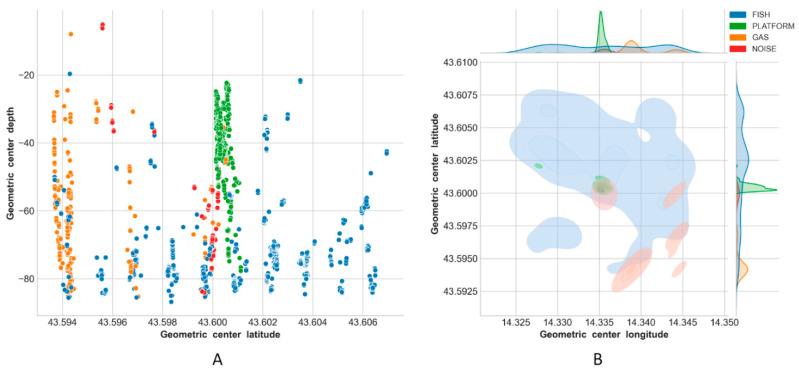
Predictions on Survey 1: latitude-depth view (**A**), and Kernel Density Estimation plot (top view) (**B**).

**Figure 14:**
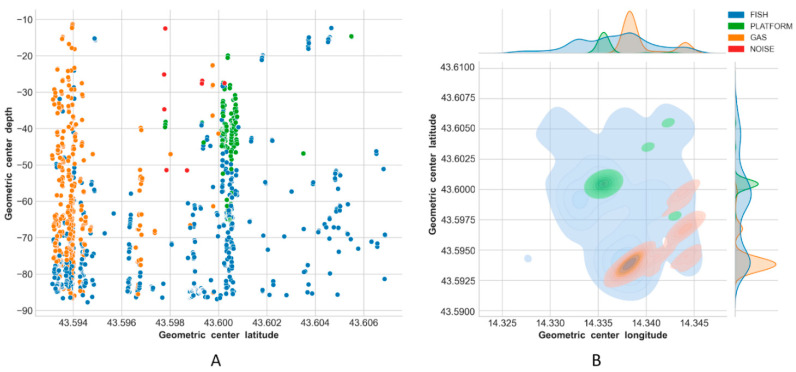
Predictions on Survey 2: latitude-depth view (**A**), and Kernel Density Estimation plot (top view) (**B**).

**Figure 15:**
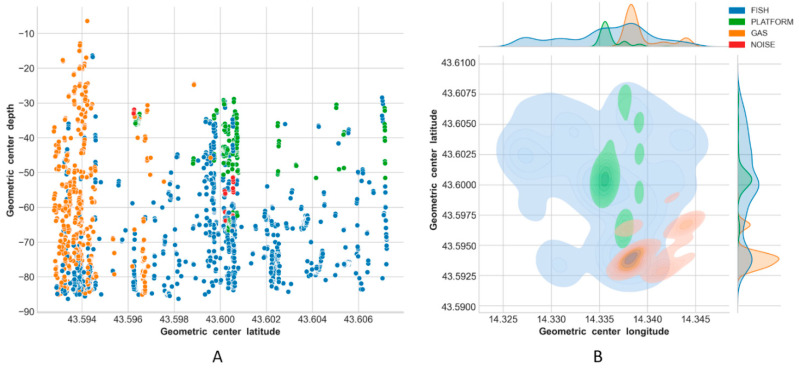
Predictions on Survey 3: latitude-depth representation (**A**), and Kernel Density Estimation plot (**B**).

**Figure 16:**
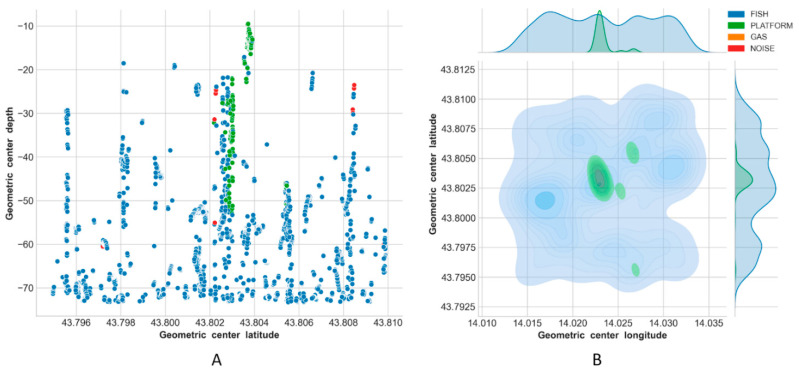
Predictions on Survey 4: latitude-depth representation (**A**), and Kernel Density Estimation plot (**B**).

Results of the classification are reported in Figure 13, Figure 14, Figure 15 and Figure 16, using latitude-depth views (panels A) and Kernel Density Estimation (KDE) top views (panels B).

Depth profiles (A panels) in Figure 13, Figure 14, Figure 15 and Figure 16 show that the platform was always correctly identified as expected at the center of the unseen scenes, especially in the upper depth layers. The bottom part of the structure was, on the contrary, often masked by the presence of fish aggregations, gas and/or noise. Gas plumes were correctly detected in the southeastern portion of the survey area surrounding Platform A, and this phenomenon was always registered with different intensities over the three related surveys (Figure 13, Figure 14 and Figure 15, Platform A). By contrast, no gas was detected in the 1500 m × 1500 m area surrounding Platform B in Survey 4 (Figure 16).

Fish aggregations were scattered throughout the entire water column in Survey 4, and mostly in the lower depths in Surveys 1–3. By contrast, the southwestern portion of the area surrounding Platform A was generally less populated by fishes, while the footprint of this class was more homogeneously distributed around Platform B.

Gas seeps were consistent in Surveys 2 and 3 in the southeastern part, while they were less pronounced in Survey 1 and not seen, as expected, in Survey 4.

For Surveys 3 and 4 in particular, the ML algorithm erroneously predicted PLATFORM for a few points scattered over the sounded area (Figure 15B and Figure 16B), while noise was always mainly located close to the platform.

## 4. Discussion and Conclusions

This work identifies a method to speed up the processing of MBES data, which often still requires a huge amount of time and the constant supervision of the operator to manually clean data and identify features to be extracted from the WCIs.

The extraction phase was performed in Echoview by way of a reproducible workflow, embedded in a template that can be efficiently applied to new surveys. After some tests, targets were extracted by opting for the “by-ping” algorithm (cutting the sounded object in slices) rather than sounding the entire object by the “cruise scanning” algorithm that can miss parts or entire objects when pings overlap in space. Nevertheless, delineation/detection of the entire target as an individual 3D object (rather than a group of slices) may further improve data processing efficiency and ML success. This possibility can be explored when software improvements are implemented to better handle this type of data.

The developed procedure saved a considerable amount of time compared to the previous manual and time-consuming approach [36] where ping subsetting and dataflow adjustments were used to extract schools and limit target loss. The next release of Echoview will include additional optimizations that were identified during this analysis, enabling even greater efficiency in future studies.

Different templates were also put in place for different water depths. Starting from the ideal assumption that the arbitrary thickness of the target slice (Figure 6B) should remain the same in order to obtain comparable results in terms of metrics, a second template was prepared for shallow waters (~10 m depth) fixing the dimension of the minimum detectable target relative to the beamwidth. This effort made the Echoview dataflow effectively exportable and applicable also to different conditions.

The intuition that the identified target classes could be characterized by well-defined features draw the design of the ML pipeline. Fish schools are often spatially confined and compact, and their N-S and E-W—as well as their Top-Bottom dimensions—are often comparable. On the other hand, gas seeps are scattered and extend from the seabed to the surface, with their Top-Bottom dimension bigger than the others (N-S or E-W). Fish schools and gas seeps also differ in terms of velocity: the first class is characterized by a low 3D velocity (lesser than 5 m/s [49]) with the bigger component of this vector parallel to the seabed, whereas the second can also reach speeds over 10 m/s [17] with the orthogonal component overwhelmingly bigger. Moreover, as the spatial distribution of gas plumes is scattered, the volume of slices is larger for fish schools than gas seeps.

ML techniques were used in a first instance to verify, from metrics data extracted, if assumptions made above on shape and behavior of fish schools and gas seeps were confirmed. With an unsupervised usage of the stacked methods it was found that depth and height played a key role in discriminating gas from fish. Surface area and bounding box dimensions of the target were also relevant in discrimination.

Where the goal of feature engineering was to transform existing features and construct new features to improve the performance of a model, feature selection was about reducing the dimensionality of the data set by removing unnecessary features. In this context, the use of features related to Sv_UNCAL_ were considered and it was opted for the creation, and following adoption, of the only derived difference between Sv_UNCAL_ maximum and mean values (Sv_UNCAL_ diff). This was also due to the consideration that sidelobe interference noise can hinder the detection of targets and affect the measure of their minimum Sv_UNCAL_ values.

After having set the feature space, training with labeled data coming from Survey 1 was necessary to implement a first semi-supervised learning. This operation allowed to utilize unlabeled data while training the ML model, give a slight performance boost and improve the accuracy of further predictors [44]. These pseudo-labels were key, given the small amount of labeled data that was available for training. It should not be underestimated in fact that training ML models for classification requires a large number of labeled samples that are usually expensive to collect in WC applications, although we still have abundant unlabeled data.

The final ensemble stack model reached an accuracy of ∼98% while making predictions on Survey 1, and gave consistent classification results for Survey 2 and Survey 3 where it was used to rapidly evaluate temporal/spatial trends over the same sounded area (Figure 13, Figure 14 and Figure 15).

Predictions on Survey 4 were performed to investigate the transferability of trained models. Results seemed to be consistent, correctly identifying the presence of the unseen platform and the absence of gas seeps that were never manually observed in this relatively small 1500 × 1500 m area surrounding Platform B (Figure 16). It leads us to believe that the adoption of the classification model at the new test site could be easily improved by including relatively small site-specific training data [31].

The lowest recall value (87%) was attributed to the NOISE class, for which the model gave the highest number of false positives (incorrectly classified as FISH).

Even if the PLATFORM class had a very high proportion of correctly identified targets (99%), such predictions did not seem to be reasonable because sometimes they were far from the real position of the structure. This could be discussed in terms of the ML performance as well as in terms of the reliability of the manual labelling itself. Noise from the extraction activities could hinder labelling in the proximity of the platform where NOISE and PLATFORM targets coexist, as well as high noise levels near the structure and systematic sidelobe artefacts in general could hamper the classification process by degrading MBES WCI and related target features.

Lastly, when speaking about model accuracy, it is important to stress that results should be interpreted by keeping in consideration three different levels of uncertainty we introduced in: (i) target extraction, due to the software that processed WCI; (ii) manual labelling, due to the operator that visually classified targets; (iii) target prediction, due to the designed ML approach.

In light of the above, ongoing research aims to enrich the feature space with bathymetry-derived features (e.g., slope, curvature, bathymetric position index [50]). These may give more or better information on hydrocarbons whose efflux through the seafloor causes positive or negative seafloor geomorphological features, such as pockmarks [16].

The reliability of the obtained results together with the efficiency of the whole data processing gain additional importance in the framework of multiannual integrated monitoring programs, such as those conducted by CNR-IRBIM that acquires, on a monthly basis, MBES data around a few offshore gas platforms to understand the role of these offshore artificial habitats and their impact on the ecology of fish populations. This always resulted in a large volume of WCI data whose processing demanded time and effort.

MBES calibration [51] could improve the ML performance by providing additional information for human labelling or machine target classification [52]. However, it must be said that the use of uncalibrated MBES for feature classification did not penalise the ML approach that mostly used features (e.g., height, dimensions, volume, speed) that connected to the recorded Sv_UNCAL_ values. Also, the use of object detection algorithms in Echoview is reasonable since the Sv_UNCAL_ mean values are not taken “as a whole” but compared in the frame of the same survey data (same instrument set-up, and same sea conditions). It is worth mentioning that besides systematic calibration of the MBES, the measurements can be disturbed by several environmental and physical factors (e.g., sea conditions, current and mud suspension dynamics, water turbidity) that can cause fluctuations of absolute high-frequency backscatter values from time to time [53,54]. This encourages the routine acquisition of different and concurrent environmental data together with such tightly spaced MBES survey data and their use to support the interpretation of trends in backscatter levels.

This study demonstrates a comprehensive and reliable approach for the detection and classification of targets in Kongsberg EM2040CD data, providing valuable insights into ecosystem and seafloor processes. The approach is transferable to data recorded by other hydrographic MBES systems that record WCI and provides an opportunity to extract more value from the investment required to perform MBES surveys.

## Figures and Tables

**Table 1:** The MBES datasets used in this paper.

Site	Survey	Date	Dataset Role	Number of Slices Detected
A	1	12-11-2018	Training + unseen	2418
A	2	24-07-2019	Unseen	1663
A	3	23-10-2020	Unseen	2554
B	4	03-06-2020	Unseen	2389

**Table 2:** Descriptions and units of the 24 metrics used for target classification.

Metric	Description	Unit of Measure
x	Flattened latitude of the center of mass of the slice	n.a.
y	Flattened longitude of the center of mass of the slice	n.a.
Geometric center latitude	Latitude of the geometric center of the slice	Decimal degrees
Geometric center longitude	Longitude of the geometric center of the slice	Decimal degrees
Mass center latitude	Latitude of the mass center of the slice	Decimal degrees
Mass center longitude	Longitude of the mass center of the slice	Decimal degrees
Geometric center depth	Depth of the geometric center of the slice	m
Mass center depth	Depth of the mass center of the slice	m
Depth ratio	Ratio between geometric and mass center of the slice	n.a.
Vertices	Number of vertices in the polyhedral object that represents the slice	n.a.
Triangles	Number of faces in the polyhedral object that represents the slice	n.a.
Height	Height of the slice (difference between maximum and minimum depth)	m
Surface area	Surface area of the polyhedral object that represents the slice	sqm
Base	Ratio between surface area and height of the slice	m
Cluster	Cluster associated to the slice from the k means algorithm	n.a.
Length 1	Longest dimension of the object-aligned bounding box (the bounding box is oriented with respect to the transect direction)	m
Length 2	Second longest dimension of the object-aligned bounding box	m
Length ratio	Ratio between the two longest dimensions of the bounding box containing the slice	n.a.
Relative depth in time	Component of the velocity vector referred to depth direction for one slice respect to the successive in the same multiping object	m/s
Sv_UNCAL_ diff	Difference between maximum and mean values of Sv uncalibrated for the slice	dB
Sample mean	Mean backscatter of the samples in the slice	dB
Beams	Number of beams that intersect with the slice	n.a.
Intersection area first beam	Intersection area between the slice and first beam in the variable used to create it in detection phase. This variable and the following provide an indication of whether the object was fully or partially insonifed by the swath	sqm
Intersection area last beam	Intersection area between the slice and last beam in the variable used to create it	sqm

**Table 3:** Classification report for the pseudo-labelling.

Class	Precision	Recall	F1-Score	Support
FISH	0.95	0.99	0.97	325
GAS	0.95	0.98	0.97	145
NOISE	1.00	0.56	0.71	36
PLATFORM	0.99	0.98	0.98	86
accuracy	-	-	0.96	592
macro avg	0.97	0.87	0.91	592
weighted avg	0.96	0.96	0.95	592

**Table 4:** Classification report for the ensemble model.

Class	Precision	Recall	F1-Score	Support
FISH	0.98	0.99	0.99	1075
GAS	0.98	0.97	0.98	265
NOISE	0.95	0.87	0.91	70
PLATFORM	0.99	0.99	0.99	283
accuracy	-	-	0.98	1693
macro avg	0.98	0.96	0.97	1693
weighted avg	0.98	0.98	0.98	1693

## Data and Code Availability Statement

MBES survey data (https://doi.org/10.17882/79142, accessed 23 April 2021) and ML code (http://doi.org/10.5281/zenodo.4621173, accessed 23 April 2021) are published and freely available to view, download, modify and reuse upon citation (license CC-BY v. 4.0).

## Appendix A

**Table A1:** Key decision table for the MBES target extraction procedure.

Processing Stage	Purpose	Description	Key Settings
Explore	Establishing general data characteristics to inform the optimal processing approach	Querying the data via echograms, graphs, tables, maps and 4D views. Maximum Intensity operator used to provide data overview	None
Calibrate	Establishing correct location of data in terms of latitude, longitude, range from transducer, and time. Backscattering strength was not calibrated in this study	GPS fixes recorded to .wcd file used as position source Heading data recorded to .wcd file used as heading source Ping Time Shift used to offset time of pings from the second head in dual beam system	Platform type: position determined by GPS Position source: “position GPS fixes” Heading source: “heading data” Pings shifted by time: 1 ms
Clean	Removal of unwanted targets, acoustical or electrical noise, and statistical variation in the measurements	Bad data regions created on Maximum Intensity variable to exclude pings with excessive noise. 3 × 3 Convolution, Erosion Filter 3 × 3, Dilation Filter 7 × 7 (×2) used to reduce stochastic variance in 2D echogram. Best Bottom Candidate Line Pick and Span Gaps used to detect seafloor in 2D echogram. Threshold Offset used to detect surface noise in 2D echogram. Data Range Bitmap, Mask, Reduce Pings, Match Ping Times, and Processed Data used to remove 3D pings and/or samples that contain no backscatter of interest in 2D echogram. XxYxZ Convolution used to reduce stochastic variance in 3D echogram. Fixed Range surface used to exclude noise at transducer face. Multibeam bottom surface used to delineate seafloor backscatter. Surface resampling used to smooth delineated seafloor	Minimum data threshold (dB): −36.0 Minimum Sv for good pick (dB): −20 Use backstep: true Discrimination level (dB): −10 Backstep range (m): −0.5 Peak threshold (dB): −40 Maximum dropouts (samples): 5 Window radius (samples): 8 Minimum peak asymmetry: −1 Threshold offset threshold (dB): −70 Apply line-relative smoothing: true Data range bitmap min. in-range value (dB): −998 Data range bitmap max. in-range value (dB): 999 XxYxZ Convolution algorithm: Top hat Rows (samples): 3 Columns (beams): 5 Layers (pings): 3 Fixed Range (m): 4–10 (data dependent) Multibeam surface triangulation distance (m): 20 Start depth for seafloor detection (m): 4–10 (data dependent) Min. threshold factor (%): 50 Min. sample gap between candidates: 15 Max. candidates per beam: 5 No. beams used for seeding: 3 No. samples to join: 20 Max. difference in range between neighbours (%): 5 Max. number of samples rejected before stopping: 8 Max. range of edge samples (%): 5 Resample north-south resolution (m): 2 Resample east-west resolution (m): 2
Detect and track	Delineation of backscattering targets in the water column and tracking of targets over multiple pings (region tracking)	3D school detection algorithm used to delineate contiguous clusters of above threshold backscatter on the Processed Data echograms Region tracking used to identify detections of the same target across multiple pings.	Detection algorithm: By ping Region width (m): 0.8 Minimum longest dimension (m): 2.0 Minimum middle dimension (m): 1.5 Minimum shortest dimension (m): 0.8 Save vacuoles: false Region tracking analysis variable: none (geometric center used) Alpha: 0.7 Beta: 0.7 Exclusion distance (m): 1.5 Weighting: distance in space, distance in time, volume all equal Minimum number of regions in a track: 1 Maximum distance gap between regions (m): 1 Maximum time gap between regions (s): 10
Characterise	Calculate metrics from the detected and filtered components of the signal	Scene > Export > Analysis by Regions by Region Track	Scene analysis variable: Merge Pings Integration algorithm: Multibeam cruise scanning—equal ping weight
